# Chemoenzymatic Preparation of a *Campylobacter jejuni* Lipid-Linked Heptasaccharide on an Azide-Linked Polyisoprenoid

**DOI:** 10.1021/acsomega.3c01657

**Published:** 2023-04-22

**Authors:** Amanda J. Reid, Katelyn M. Erickson, Joseph M. Hazel, Vinita Lukose, Jerry M. Troutman

**Affiliations:** † Nanoscale Science Program, University of North Carolina at Charlotte, 9201 University City Blvd., Charlotte, North Carolina 28223, United States; ‡ Department of Chemistry, University of North Carolina at Charlotte, 9201 University City Blvd., Charlotte, North Carolina 28223, United States; § Department of Chemistry, The Ohio State University, 281 W Lane Avenue, Columbus, Ohio 43210, United States; ∥ Departments of Chemistry and Biology, Massachusetts Institute of Technology, 77 Massachusetts Avenue, Cambridge, Massachusetts 02139, United States

## Abstract

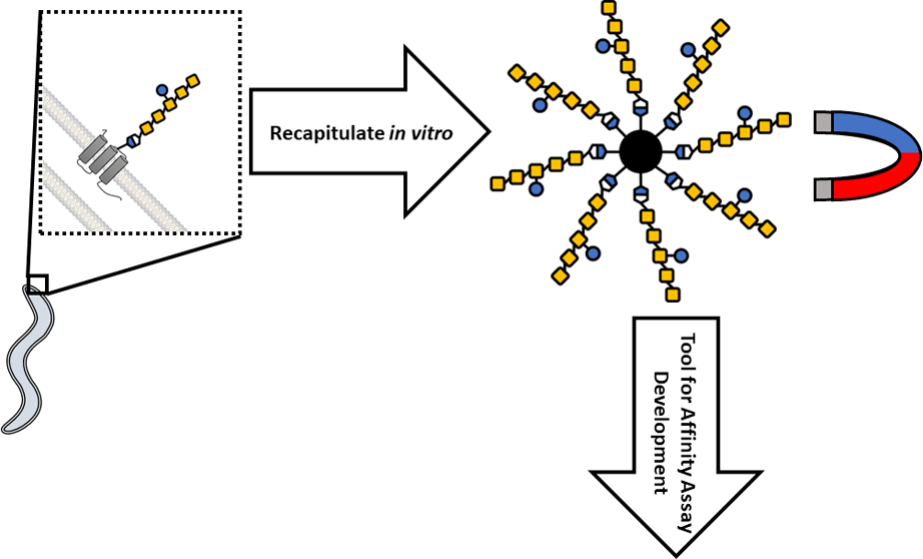

Complex poly- and oligosaccharides on the surface of bacteria provide a unique fingerprint to different strains of pathogenic and symbiotic microbes that could be exploited for therapeutics or sensors selective for specific glycans. To discover reagents that can selectively interact with specific bacterial glycans, a system for both the chemoenzymatic preparation and immobilization of these materials would be ideal. Bacterial glycans are typically synthesized in nature on the C55 polyisoprenoid bactoprenyl (or undecaprenyl) phosphate. However, this long-chain isoprenoid can be difficult to work with in vitro. Here, we describe the addition of a chemically functional benzylazide tag to polyisoprenoids. We have found that both the organic-soluble and water-soluble benzylazide isoprenoid can serve as a substrate for the well-characterized system responsible for *Campylobacter jejuniN*-linked heptasaccharide assembly. Using the organic-soluble analogue, we demonstrate the use of an *N*-acetyl-glucosamine epimerase that can be used to lower the cost of glycan assembly, and using the water-soluble analogue, we demonstrate the immobilization of the *C. jejuni* heptasaccharide on magnetic beads. These conjugated beads are then shown to interact with soybean agglutinin, a lectin known to interact with *N*-acetyl-galactosamine in the *C. jejuni* heptasaccharide. The methods provided could be used for a wide variety of applications including the discovery of new glycan-interacting partners.

## Introduction

The bacterial cell surface is decorated with a variety of complex sugar coatings from modifications to lipopolysaccharides including O-antigens to capsular polymers and glycosylated proteins.^1−4^ These glycans can play important roles in pathogenic and symbiotic interactions with hosts and in the survival of the organism in inhospitable environments.^5−10^ Advances in chemical and analytical tools have highlighted the role of bacterial surface glycans in pathogenicity, cell surface adhesion, and biofilm formation.^11−14^

Bacterial surface glycans often differ from one species to another and even between sub-species with over 600 different monosaccharides possible throughout the prokaryotic world.^15,16^ Therefore, methods to selectively sense these glycans could provide a means to differentiate bacteria based solely on surface glycan presentation. Developing sensors for complex bacterial glycans often depends on immobilization of target glycoconjugates to enrich high-affinity binding partners.^17^ Immobilized glycans could also be used to evaluate the specificity of glycan-interacting proteins, develop microarrays,^18^ and synthesize glycans on the solid phase.^19^

**Figure 1:**
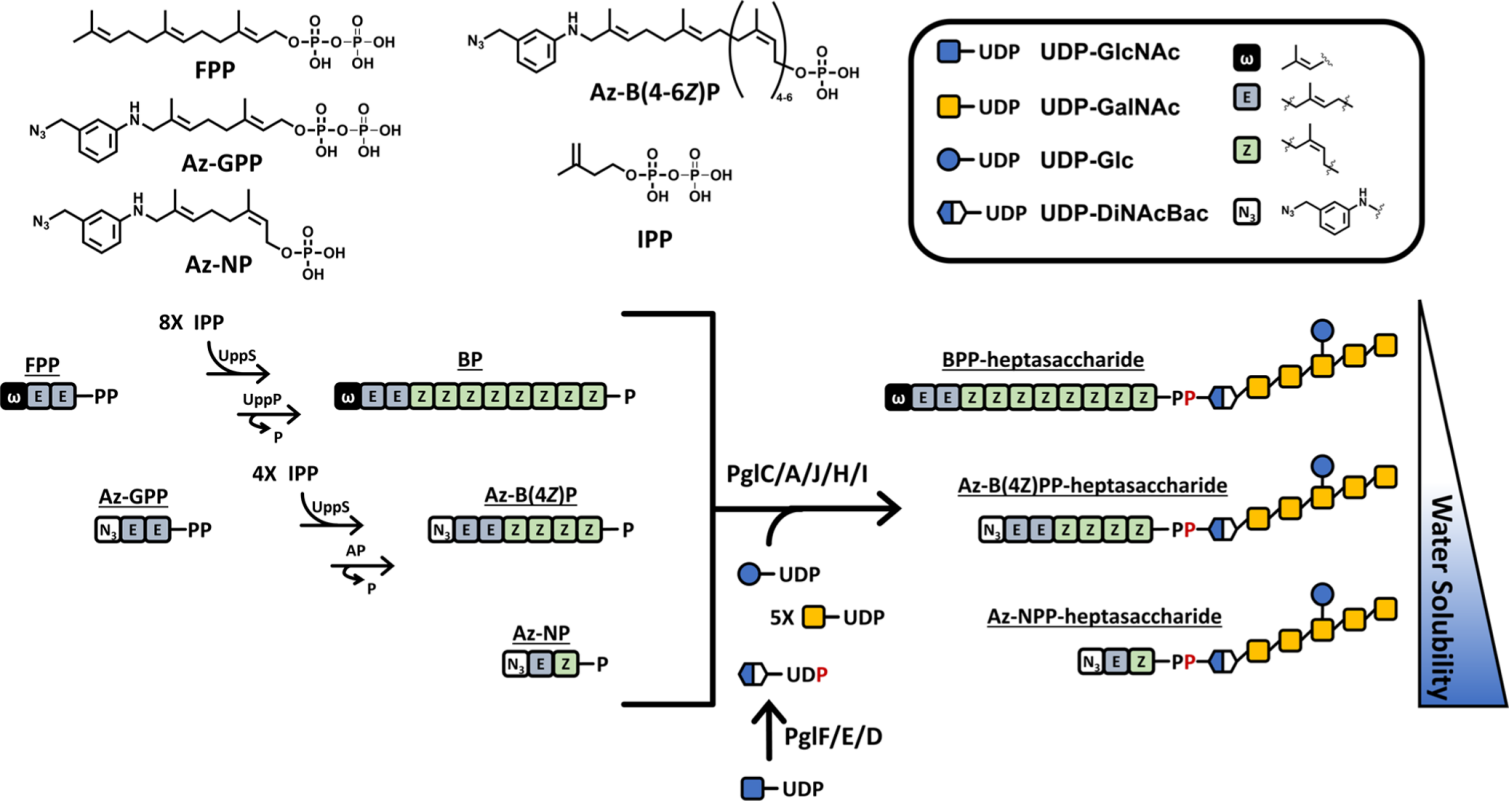
Assembly of protein glycosylation (Pgl) oligosaccharides from *Campylobacter jejuni* (*C. jejuni*) with native BP and isoprenoid tags used in this work. Native BP is produced from the cis condensation by undecaprenyl pyrophosphate synthase (UppS) and subsequent dephosphorylation by undecaprenyl pyrophosphate phosphatase (UppP). Polyisoprenoid tags are prepared by a similar route by tuning the length and utilizing potato acid phosphatase (AP). Alternatively, a two-isoprene monophosphate probe was synthesized directly. Sequential glycan addition is then achieved by the transferase enzymes PglC, PglA, PglJ, PglH, and PglI from the *C. jejuni* Pgl pathway with their respective sugar substrates.

Enzymatic methods for complex glycan synthesis exploit the natural specificity and selectivity of enzymes to avoid more complex synthetic schemes. In nature, bacteria often build glycans on the membrane-embedded C55 anchor bactoprenyl phosphate (BP, also called Und-P) (Figure 1).^20^ BP is synthesized in bacteria through the condensation of eight isopentenyl diphosphates (IPP) with a single C15 farnesyl diphosphate (FPP) by undecaprenyl pyrophosphate synthase (UppS). The C55 diphosphate product is then dephosphorylated by an undecaprenyl pyrophosphate phosphatase (UppP) to give BP. Our group has taken advantage of the natural dependence of glycan assembly on this lipid anchor to chemoenzymatically prepare important bacterial glycans on fluorescently tagged BP analogues.^21−23^ Recently, the Cochrane group described the development of polyisoprenoid analogues prepared semi-synthetically.^24^ Of particular interest from that work was the addition of an azide functionality that provides a ready functional handle for conjugating glycans formed on a polyisoprenoid through a wide range of click-chemistry-enabled materials.

In this work, we develop a method for glycan assembly and immobilization using the heptasaccharide (Figure 1) that is conjugated to proteins from the food-borne pathogen *C. jejuni*. The heptasaccharide can attenuate the ability of the organism to colonize a host.^4,25,26^ This system is ideal for developing an immobilization system amenable to chemoenzymatic synthesis because it is very well characterized both in vitro and in vivo.^4,27−30^ The chemical structure of the glycan has been clearly documented, and the identity and function of the enzymes involved in its construction are well understood. Finally, the high *N*-acetylgalactosamine (GalNAc) content of the final heptasaccharide facilitates detection via the glycan-interacting lectin, soybean agglutinin (SBA).^26^

## Results

### Preparation of the Lipid Acceptor for *C. jejuni* Heptasaccharide Synthesis

**Figure 2:**
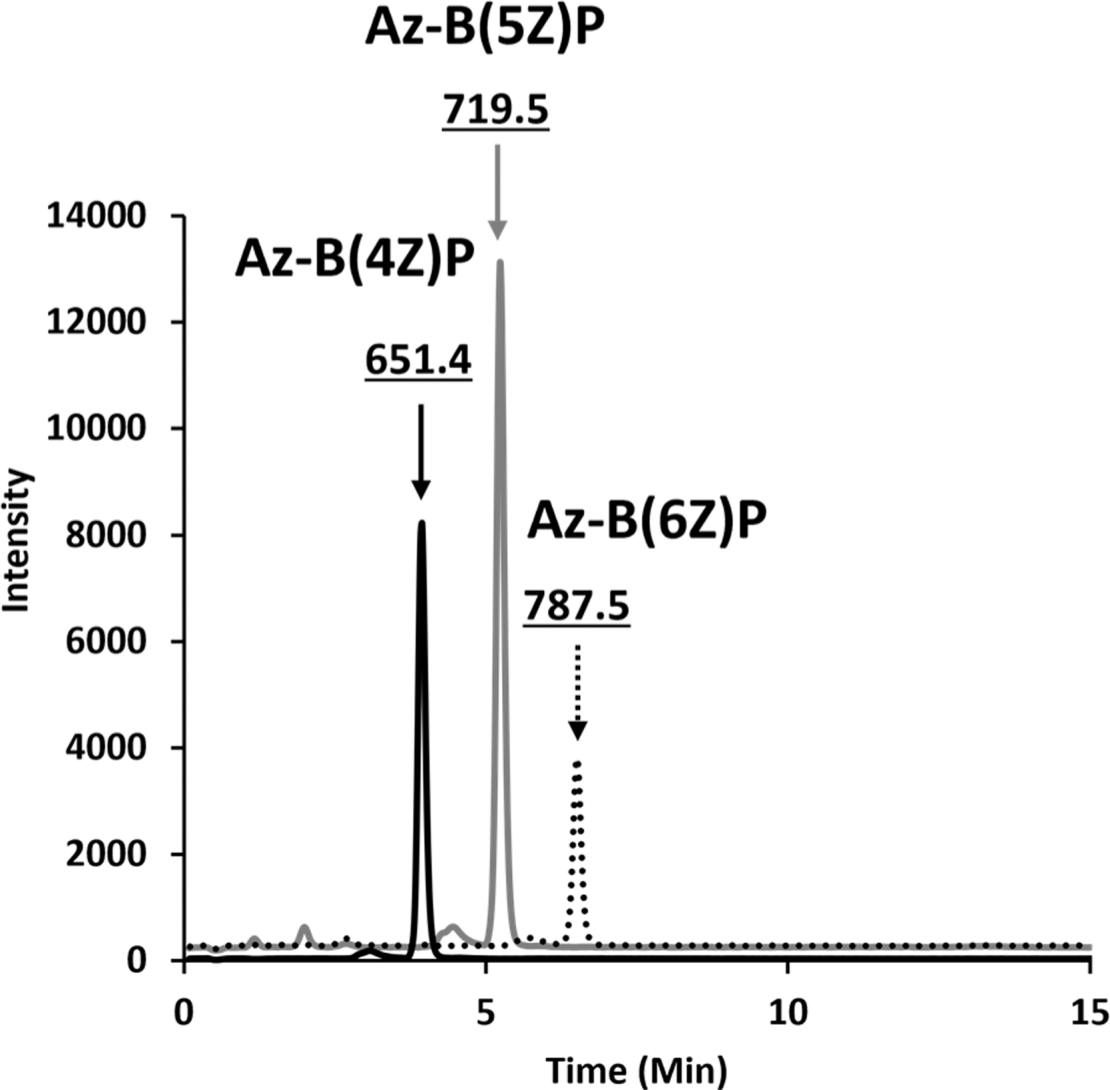
Enzymatic synthesis of benzylazide BP LC–MS of Az-BP product formation by UppS and potato acid phosphatase. SIM analysis of [M-1H]^−1^ ion species in separate channels corresponding to Az-BPs from 4Z-6Z (651.4, 719.5, and 787.5 *m*/*z*, respectively, where 4–6 denote the number of Z configuration isoprenes incorporated by UppS.

Our goal was to develop a system to chemoenzymatically prepare glycans that could readily be immobilized for the detection of glycan-interacting partners. To do this, we first focused on the assembly of an azide-linked polyisoprenoid that could serve as the lipid anchor for enzymatic glycosylation reactions. To incorporate an azide into BP, we first synthesized a benzylazide-linked geranyl diphosphate (Az-GPP, Figure 1), which served as an FPP analogue with the terminal isoprene (ω) replaced with the benzylazide.^21−23,31−33^ We then tested whether this compound could serve as a substrate for UppS and whether it could be readily dephosphorylated to provide a benzylazide BP (Az-BP, Figure 1).^22^ Previous analogues developed by our group included fluorescent tags which were easily monitored by high-performance liquid chromatography (HPLC) and fluorescence detection. However, the benzylazide was not fluorescent and its molar absorptivity was relatively low, which prompted us to utilize liquid chromatography–mass spectroscopy (LC–MS) for product detection. Using LC–MS selective ion mode (SIM) analysis of a UppS and potato acid phosphatase reaction with Az-GPP, we found that we readily formed Az-BP products with 4–6 *Z-*configuration isoprene additions (4-6*Z*) (Figure 2). To test the ability to use the benzylazides in a conjugation reaction and to enhance detection capability without mass spectrometry, we performed a model reaction with TAMRA-linked dibenzocyclooctyne (DBCO), the Az-GPP starting material, and the enzymatic products. The two isomers of TAMRA conjugated to Az-GPP were readily apparent in these reactions when analyzed by HPLC. The isomers were poorly resolved with the Az-BPP and Az-BP products of UppS and potato acid phosphatase (Figure S1).

### Building the Pgl Heptasaccharide on Click-Enabled BP

Our next focus was on the preparation of a glycan that could be used as a model system for glycan binding partner detection. We chose the *C. jejuniN*-linked heptasaccharide as our model because all enzymes required for its formation have been identified and studied extensively and its structure has been well characterized.^4,29,30,34^ The biosynthesis of the *C. jejuni* heptasaccharide begins with the production of a uridine diphosphate (UDP)-linked *N*,*N*-diacetylbacillosamine (diNAcBac) by the enzymes PglF, PglE, and PglD.^34^ The product of PglD, UDP-diNAcBac, then serves as a diNAcBac-phosphate donor for transfer to BP by the enzyme PglC.^29,30^ The UDP-diNAcBac substrate for PglC was prepared using published procedures, and product formation was analyzed by HPLC (Figure S2) utilizing a column with an amine stationary phase.^34,35^ The identity of the product was confirmed by mass spectrometry.

**Figure 3:**
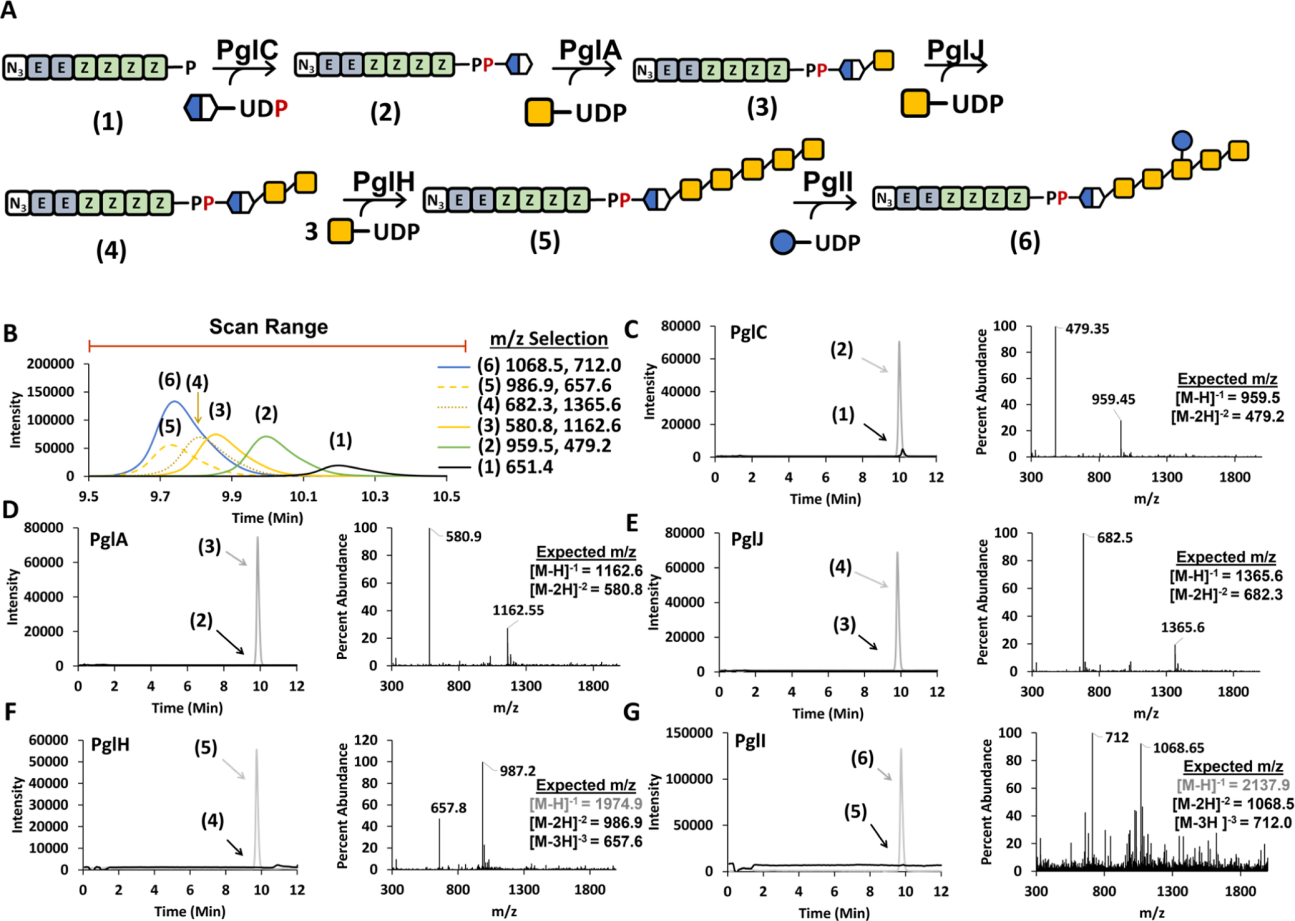
LC–MS analysis of sequential Pgl oligosaccharide formation on Az-B(4Z)P. (A) Biosynthesis pathway to the *C. jejuni* heptasaccharide. (B) Selective ion mode (SIM) chromatograms for each product indicated in A using the *m*/*z* shown for each. The chromatogram in B is an overlay of six different analyses. The scan range is indicated for total ion plots in C–G. (C) PglC reaction with Az-B(4Z)P (1) and UDP-diNAcBac. SIM chromatograms are shown for the starting material (black) and product (gray) after the reaction. Total ion plot provided for scan range indicated in B. (D) Same as C with PglA and UDP-GalNAc. (E) Same as D with PglJ. (F) Same as E with PglH. (G) Same as F with PglI and UDP-glucose. All analyses were performed on single-pot reactions.

Our group has previously shown that fluorescent analogues of BP can be utilized by the enzymes PglC and PglA of the *C. jejuni* heptasaccharide assembly system.^36,37^ However, modification of the terminal isoprene could impact the ability of these isoprenoids to serve as substrates for these enzymes. To test this PglC, PglA, PglJ, PglH, and PglI were prepared as previously described (Figure S3).^30^ Reactions mixtures were then prepared with UDP-diNAcBac, HPLC purified benzylazide-linked BP with four isoprene additions (Az-B(4Z)P, 1) and PglC (Figure 3A). Product formation for the PglC reaction (2) was then monitored by LC–MS using SIM detection of −1 and −2 charged species for the product and the −1 charged species for the Az-B(4Z)P (1) starting material (Figure 3B). In this reaction, we observed nearly complete depletion of the starting material with the formation of a new peak with an *m*/*z* 959.5 and 479.2 (Figure 3C). The total ion spectrum of the new product peak indicated that the major component of this peak was the −1 and −2 charged species of the Az-B(4Z)PP-linked diNAcBac (2) (Figure 3C). We next repeated this process with PglA, PglJ, PglH, and PglI with excess UDP-GalNAc and UDP-Glc present. Using SIM detection of the starting material and expected product (3–6), we observed nearly complete consumption of the starting material in each reaction and the formation of the product consistent with the activity of each enzyme. Each glycan addition (Figure 3A) resulted in products with a characteristic retention time, eluting at 9.99 (2), 9.85 (3), 9.80 (4), 9.72 (5), and 9.73 (6) min (Figure 3B). Total ion plots were generated over the full elution range (9.5–10.5 min) for each product to confirm that the BPP-linked sugar product was the major product of each reaction (Figure 3D–G). The dominant *m*/*z* observed was the −2 charged species for each except the PglI product (6) where both the −3 and −2 species were detected to the same extent. The −1 charged species for the PglH and PglI products was not observed as these would surpass the mass limits of our MS detector.

### In Situ UDP-GlcNAc Epimerization by WbpP from *Vibrio vulnificus*

**Figure 4:**
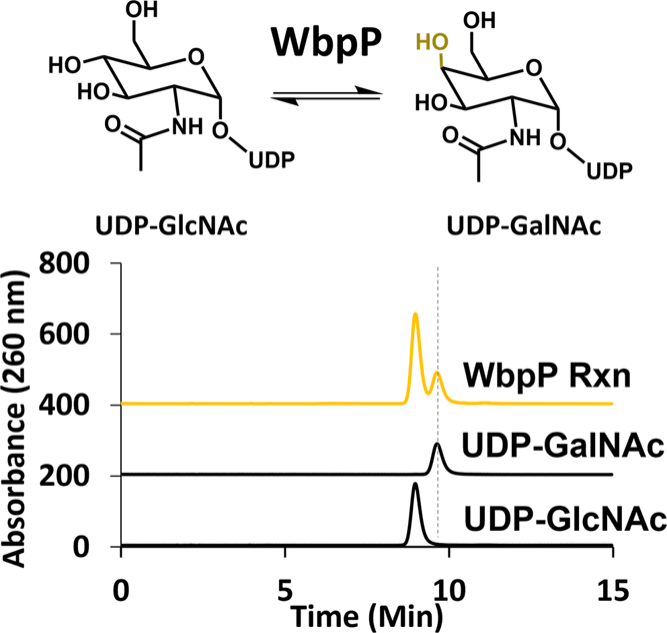
WbpP-catalyzed epimerization of UDP-GlcNAc. Overnight activity of the putative epimerase WbpP producing a mixture of UDP- GalNAc and -GlcNAc (3:7). Chromatograms were offset along the *y*-axis by 200 units.

One important issue in the assembly of the *C. jejuni* glycan was the high cost associated with UDP-GalNAc. UDP-GalNAc is more than ten times the cost per milligram of UDP-*N*-acetylglucoseamine (GlcNAc), and considering that five GalNAc residues are incorporated into the *C. jejuni* glycan, this could be cost prohibitive. Methods are available to enzymatically synthesize UDP-GalNAc.^38^ We instead tested if a GlcNAc epimerase could be exploited to produce UDP-GalNAc from UDP-GlcNAc in situ. We cloned and expressed the *V. vulnificus* putative *N*-acetyl-hexosamine (HexNAc) 4-epimerase *WbpP*.^39,40^ In the presence of UDP-GlcNAc, purified WbpP afforded a product with an identical retention time as a UDP-GalNAc standard at 9.6 min by HPLC (Figure 4). The equilibrium of an overnight reaction yielded a product ratio of 3:7 (UDP-GalNAc/UDP-GlcNAc). This relative equilibrium was identical to that reported for a *Pseudomonas aeruginosa* WbpP, which has 70% identity to the *V. vulnificus* WbpP.^39^

### Monitoring the Specificity of Glycosyltransferases with a Benzylazide Bactoprenyl Analogue

**Figure 5:**
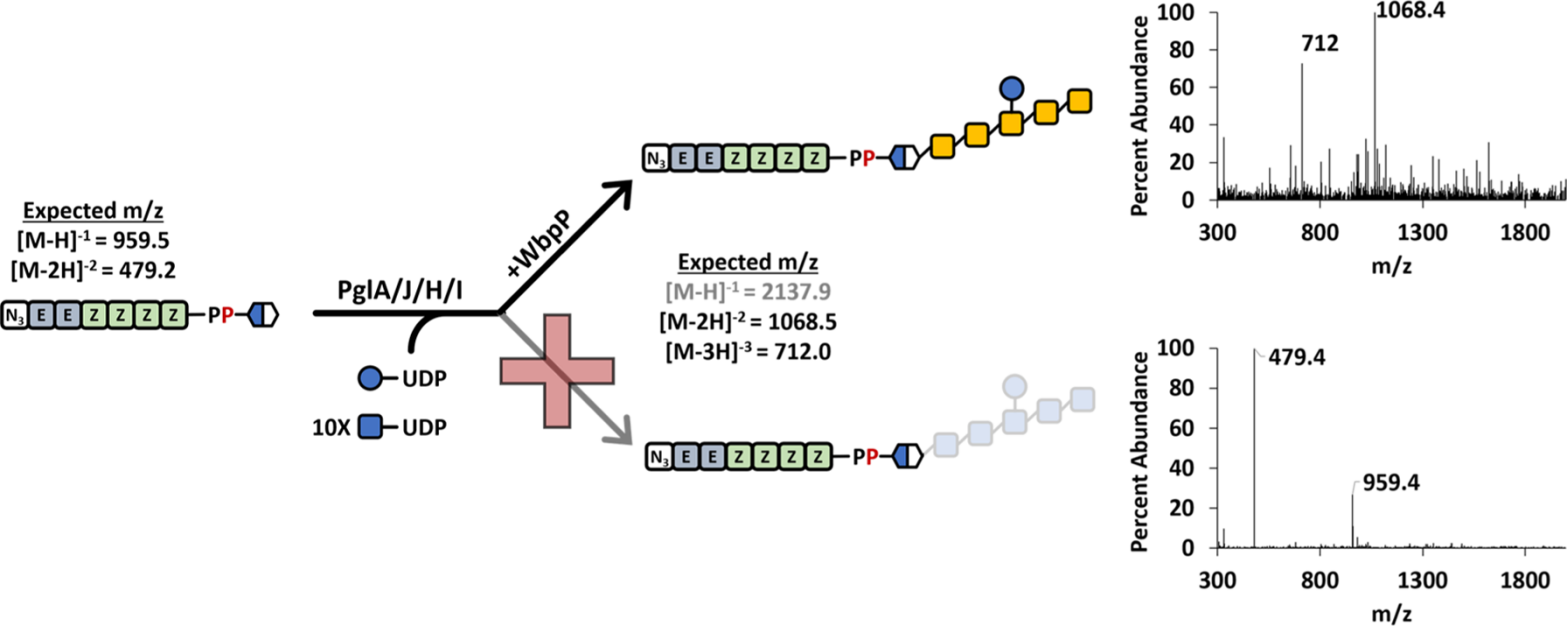
Glycan assembly with in situ generation of UDP-GalNAc. Pgl heptasaccharide formation only occurs with the addition of WbpP as PglA, PglJ, and PglH lack activity with UDP-GlcNAc. The mass spectrum shown was for a single-pot reaction to the final product. Note that Figure S4 shows no product with each intermediate and UDP-GlcNAc.

We expected that in situ UDP-GalNAc production could be driven by the depletion of the nucleotide-linked sugar as the Pgl heptasaccharide is assembled.^41^ However, it was not clear if the Pgl HexNAc transferases A, J, or H could utilize UDP-GlcNAc as a substrate, which could confound the analysis of products formed in the presence of excess UDP-GlcNAc. Both HexNAc products have identical *m*/*z* values and are therefore indistinguishable from one another by mass spectrometry. Additionally, an excess of UDP-GalNAc was optimal for PglA, PglJ, and PglH product formation. To test the selectivity of PglA, PglJ, PglH, and PglI, we prepared Az-B(4Z)PP-diNAcBac and isolated it by HPLC. Next, we mixed this substrate with UDP-GlcNAc and observed no product formation by LC–MS (Figure S4). We then added purified WbpP and observed product formation. To test the next enzyme in the pathway and avoid purification of each isoprenoid-linked material, we took advantage of the *n-*butanol solubility of the Az-B(4Z)PP-linked sugars and extracted the product away from the water-soluble nucleotide-linked sugars. After solvent removal, we tested whether the Az-B(4Z)PP-diNAcBac-GalNAc product could then serve as an acceptor for GlcNAc and PglJ. Once again, no product was observed until this reaction was treated with WbpP. The method was then repeated with PglH, and we confirmed that PglA, PglJ, and PglH would not transfer GlcNAc. We did a similar test with PglI to ensure that it would not transfer any excess GlcNAc from UDP-GlcNAc, and as expected, it also did not (Figure S4). The complete heptasaccharide was formed on the benzylazide-linked isoprenoid in the presence of WbpP and UDP-GlcNAc (Figure 5). The organic solubility of the isoprenoid was key to this procedure and saved considerable time that would have been consumed by HPLC purification of each intermediate for testing sugar specificity.

### Az-Neryl Monophosphate Is a Substrate for Pgl Assembly

**Figure 6:**
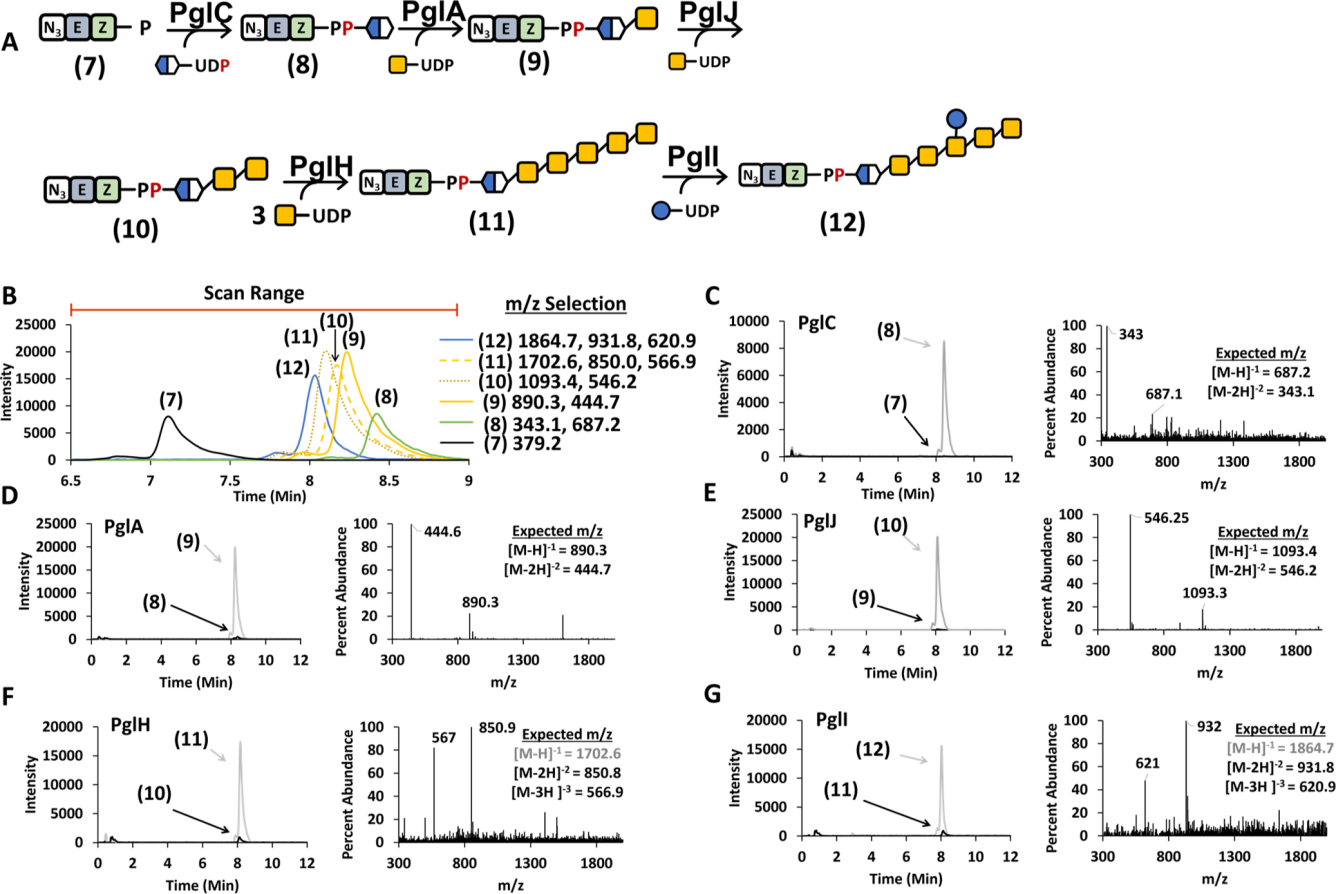
LC–MS analysis of sequential Pgl oligosaccharide formation on Az-NP. (A) Biosynthesis pathway to the *C. jejuni* heptasaccharide with Az-NP (7). (B) Selective ion mode (SIM) chromatograms for each product indicated in A using the *m*/*z* shown for each. The chromatogram in B is an overlay of six different analyses. The scan range is indicated for total ion plots in C–G. (C) PglC reaction with Az-NP (7) and UDP-diNAcBac. SIM chromatograms are shown for the starting material (black) and product (gray) after the reaction. Total ion plot provided for the scan range indicated in B. (D) Same as C with PglA and UDP-GalNAc. (E) Same as D with PglJ. (F) Same as E with PglH. (G) Same as F with PglI and UDP-glucose. All analyses were performed on single-pot reactions.

Naturally abundant *E*/*Z* isoprenoids, such as nerol, have been used for bacterial glycan production with varying degrees of success.^42−44^ We were next interested in whether a benzylazide neryl phosphate (Az-NP, Figure 1) with the critical *Z-*configuration isoprenoid in the α position adjacent to the phosphate group could be used as an effective replacement for BP. The Az-NP would omit the need for both UppS and potato acid phosphatase. Az-NP was synthesized using protocols similar to previous procedures for geranyl analogues.^31,33,45^ We next evaluated the assembly of the *C. jejuni* heptasaccharide with Az-NP as the acceptor. The formation of the heptasaccharide on Az-NP (Figure 6A) was monitored by LC–MS with stepwise addition of Pgl enzymes (Figure 6B). The first phosphosugar addition resulted in an unexpected later retention time shift from 7.11 to 8.21 min for Az-NP and the PglC product (8). After the PglC step, each new product afforded a relative retention time shift similar to the longer chain isoprenoid with retention times of 8.41, 8.23, 8.10, 8.18, and 8.03 min for subsequent Pgl enzyme products (9–12, Figure 6B). As described for Az-B(4*Z*)P, SIM was carried out for targeted masses of the starting material and product (Figure 6C–G), indicating near complete turnover to the product by each enzyme, and a scan of the elution range confirmed that the major component of each reaction was the expected isoprenoid-linked glycan (Figure 6C–G). The Az-NP analogue was considerably easier to work with and avoided the lost material in the UppS and potato acid phosphatase reactions.

### Magnetic Bead Immobilization and Detection of Neryl-Tagged Pgl Heptasaccharide

**Figure 7:**
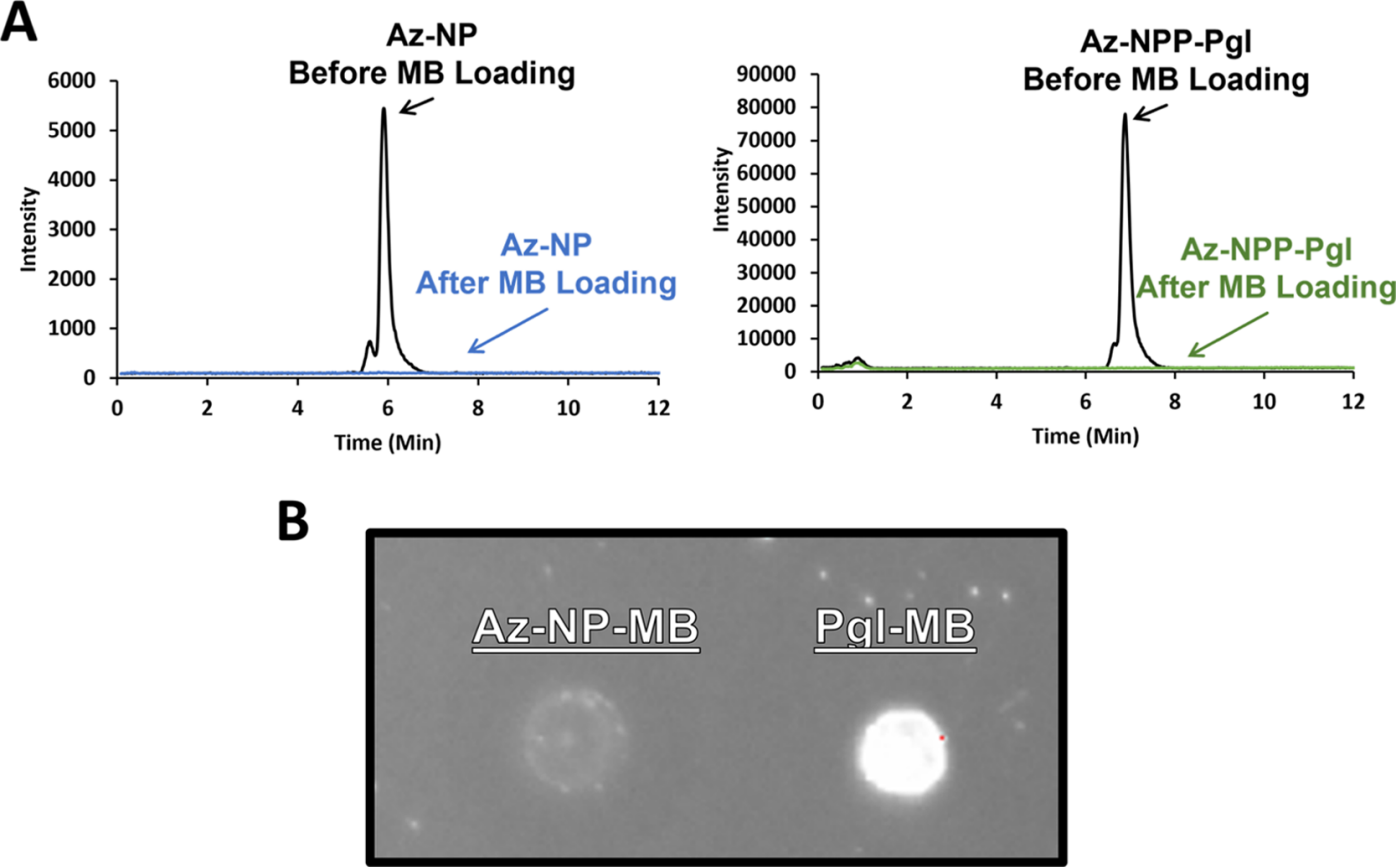
Pgl heptasaccharide immobilization onto magnetic beads. (A) LC–MS analysis of Pgl oligosaccharides on Az-NP present in the supernatant before and after introducing DBCO-coated magnetic beads (MB). (B) SBA lectin assay with Az-*N*P-MB versus Pgl-MB.

We next tested whether the Az-NPP-heptasaccharide (12) could be used for immobilization and detection of a glycan-interacting protein. To do this, we conjugated the azide to magnetic beads (MB) with surface-functionalized DBCO, providing the click-companion for copper-free “Click” addition. Unpurified reactions containing Az-NPP-heptasaccharide (12) were used directly for click-addition producing Pgl-MB. Similarly, a control bead was prepared from Az-NP (Az-*N*P-MB). The supernatant was decanted and analyzed by LC–MS to monitor the unbound oligosaccharide or isoprenoid before and after incubation (Figure 7A). The absence of either material in the supernatant following incubation suggested consumption of the azide through conjugation to the magnetic bead surface.

With the glycan immobilized on the magnetic beads, we next tested whether this material could be used for the detection of a binding interaction between the glycan and an interacting protein. We employed a fluorophore-conjugated SBA lectin used previously to detect the *N*-linked glycan on the surface of *C. jejuni*. SBA has specificity for terminal GalNAc.^4,26,46,47^ Pgl-MB and Az-*N*P-MB were first incubated with BSA to bind sites that non-specifically interact with proteins. The SBA fluorescent lectin was next incubated with the beads and then decanted and washed to remove unlinked aqueous soluble sugars. An aliquot of the beads was placed on nitrocellulose paper and imaged (Figure 7B). A bright fluorescent signal was observed at the spot containing Pgl-MB, but not Az-*N*P-MB, consistent with the presence of GalNAc moieties in the Pgl heptasaccharide and demonstrating that the beads could be used to bind and detect an interaction with the carbohydrate-interacting protein.

## Discussion

Fluorescent isoprenoid probes have aided in the identification of sequential enzyme roles during polysaccharide biosynthesis for multiple oligosaccharides.^21,23^ Here, we expand the functionality of isoprenoid-based probes by appending a bio-orthogonal handle, which greatly enhances the range of subsequent downstream applications. To illustrate this, a well-characterized heptasaccharide important for virulence in the human pathogen *C. jejuni* was recapitulated on an azide-modified isoprenoid scaffold. Furthermore, the size of the isoprenoid was reduced to the commercially available nerol, thereby affording a more direct synthesis compared with the elongated isoprenoid counterpart. The addition of a neryl probe greatly streamlines isoprenoid tag development since it can be synthesized directly, thus eliminating the need for enzymatic preparation and purification of mixed polyisoprenoids.

## Experimental Procedures

### General

Azide-labeled isoprenoids were synthesized following procedures from Labadie et al.^33^ Az-B(4*Z*)P preparation followed previously reported procedures by our group for similar analogues.^22,48^ All reagents were of ACS grade or higher. TAMRA DBCO (Sigma-Aldrich 760773), DBCO magnetic beads (Jena Bioscience CLK-1037-1), and SBA 594 conjugate (Thermo Fisher L32462) were purchased from indicated suppliers.

### Cycloaddition of Azido Isoprenoids

Azide-modified isoprenoids (neryl, geranyl, or BP/BPP) were labeled with one equivalent of TAMRA-DBCO (100 μM) in either water or UppS/potato acid phosphatase reaction conditions directly. Reactions were typically complete in under 60 min. Products were analyzed after this time on an Agilent 1100 HPLC system (Agilent Eclipse XBD-C18, 3.5 μM, 4.6 × 50 mm) monitoring for the TAMRA fluorophore (454/525 ex/em). A gradient method was used to separate BPPs and BPs with 100 mM ammonium bicarbonate (A) and *n*-propanol (B). Line B was increased from 15 to 95% over 36.9 min and then held at 95% until 42 min. LC–MS analysis of non-conjugated azide materials was performed on an Agilent 1260 LC and 6000 series ESI-MS single quad with four channels for monitoring selected ions (Waters XBridge Peptide BEH C18, 3.5 μM, 4.6 × 50 mm). *n*-Propanol was increased at a rate of 4% per min, starting at 20% with 80% of a 0.1% ammonium hydroxide solution as the aqueous component. Mass values for Az-BPs (1Z-10Z, where *Z* is the number of *Z-*configuration isoprene additions) were scanned following potato acid phosphatase treatment.

### Sugar-Modifying Enzyme Preparation and Analysis

The Pgl sugar-modifying enzymes PglF, PglE, and PglD were prepared identically to previous reports, without the addition of Triton X-100.^34^ The preparation of UDP-diNAcBac was performed in a total volume of 4 mL with 50 mM Tris-Acetate pH 7.5, 50 mM NaCl, 5 mM UDP-GlcNAc, 4.0 μM PLP, 15 mM l-glutamate, and 6 mM acetyl coenzyme A. PglF, PglE, and PglD (25 μm each) were added sequentially, and an aliquot was taken for HPLC analysis after incubation at 37 °C for 1 h. The reaction mixture was then filtered (30 K MWCO) and dried under vacuum in a centrifugal evaporator. This crude solution was then resuspended in 400 μL of water and used as the sugar donor source for subsequent Pgl assembly.

WbpP was cloned from *V. vulnificus* MO6-24 into a pET-24a vector with primers outlined in Table S1. An overnight culture of BL21-RP transformants was used to inoculate 0.5 mL of TB (10 g of tryptone, 12 g of yeast extract, and 2 mL of glycerol). The culture was grown at 37 °C with shaking until the OD reached 0.6; then, the temperature was decreased to 25 °C. IPTG (1 mM) was added, and the culture was allowed to induce for 4 h or overnight. Pelleted cells were lysed in WbpP-buffer (50 mM Tris–HCl pH 8, 200 mM NaCl) with 20 mM imidazole, and the viscous liquid was pelleted at 10,000 RCF for 30 min at 4 °C. The supernatant was then passed through 2 mL of Ni-NTA agarose and washed in WbpP-buffer with 50 mM imidazole and finally eluted in WbpP-buffer with 500 mM imidazole. Elutions containing protein were collected and dialyzed three times in WbpP-buffer.

HPLC analysis of all sugar modification reactions occurred on an Agilent 1100 monitoring at an absorbance of 260 nm (Agilent Zorbax NH_2_, 5 μm, 4.6 × 250 mm). All sugar-modifying reactions were monitored with an isocratic method and mobile phase of 200 mM ammonium acetate at pH = 4.5.

### Protein Expression of Pgl Transferase Enzymes

BL21 Star cells were transformed with each *Pgl* plasmid and used for protein expression. Overnight cultures were used to inoculate 0.5 L of autoinduction media (10 g of tryptone, 12 g of yeast extract, 2 mM MgSO_4_, 2.5 mL of glycerol, 0.25 g of glucose, 1 g of lactose, and 100 mM phosphate buffer pH 7.4) with 100 μg/mL kanamycin. Cultures were grown at 37 °C with generous shaking at 300 rpm for 4 h; then, the temperature reduced to 20 °C for 24 h. The expression of PglI was enhanced by the addition of 3% ethanol in growth media.^49^ Pelleted cultures were then lysed and purified under identical conditions to previous reports and analyzed via SDS-PAGE and western blot.^29,30,50^

### Pgl Oligosaccharide Assembly on Azide-Linked Isoprenoids

The reactions were carried out in a total volume of 40 μL with 50 mM Tris-Acetate pH 7.5, 1 mM MgCl_2_, and 100 μM tagged isoprenoid (Az-B(4*Z*)P or Az-NP). Glycans were then added at a final concentration of 200 μM UDP-diNAcBac, 2 mM UDP-GalNAc, and 1 mM UDP-Glc. Triton-extracted Pgl enzymes (8 μg/mL each) were added sequentially, and no additional Triton was added (a final concentration of 0.2% accounting for carryover). The reaction was proportionally scaled up to 500 μL and filtered (MWCO 10,000) prior to magnetic bead immobilization. When WbpP was used for in situ generation of UDP-GalNAc, reactions contained 1 μM WbpP and 5-fold excess UDP-GlcNAc relative to BP or NP. Enzyme activity was monitored by LC–MS on an Agilent 1260 LC and 6000 series ESI-MS. Either the column (Waters XBridge Peptide BEH C18, 3.5 μM, 4.6 × 50 mm) was connected to the mass spectrometry detector, for azide-tagged materials, or the column eluent was split with a TEE connector between the mass spectrometry and fluorescence detectors (2:1 split), for TAMRA-tagged materials. A gradient method was used with 0.1% ammonium hydroxide (A) and *n*-propanol (B). For Az-NP, line B was increased from 5 to 15% over 10 min. For Az-B(4*Z*)P, line B was increased from 15 to 30% over 10 min.

### PglA, PglJ, PglH, and PglI Glycan Specificity Assay

Pgl intermediates were produced as described in the previous section with Az-B(4*Z*)P as the substrate. Overnight reactions were extracted in an equal volume of *n*-butanol three times and dried under a gentle stream of air to prepare intermediates for subsequent reactions. The dried material was resuspended in Pgl reaction buffer, as described above, with 5 mM UDP-GlcNAc in place of -GalNAc. Prepared reactions for in situ generation of UDP-GalNAc additionally contained 1 μM WbpP.

### DBCO Magnetic Bead Surface Modification and Detection

Magnetic beads (0.8 mg) functionalized with DBCO were washed with water three times. A crude reaction mixture containing either Pgl heptasaccharide or Az-NP (40 nmol each) was added to the washed beads. The mixture was incubated at room temperature overnight with gentle agitation. The magnetic resin was decanted, and the supernatant was reserved for LC–MS analysis. The Pgl-functionalized beads were then washed with water three times, resuspended at 1 mg/mL, and then stored at 4 °C. For lectin binding assays, 5 μL of modified beads was blocked with 3% BSA for 30 min and then washed with water. The beads were then resuspended in 200 μL of 0.1 μg/mL of SBA 594 conjugate for two h prior to three water washes, 5 min each. All steps occurred with spinning on an end-over-end rotator at room temperature. The beads were magnetically decanted, then briefly spun, and reconstituted in the residual water following centrifugation (∼5 μL). The solution was transferred to dry nitrocellulose and imaged.

## Supporting Information Available

HPLC analysis of TAMRA-labeled benzylazide geranyl and bactoprenyl diphosphate and benzylazide BP, HPLC analysis of PglF/E/D reactions, SDS-PAGE and western blot analyses of isolated proteins, LC–MS of HexNAc specificity assays without WbpP, primers for WbpP, and synthetic production and characterization of Az-NP and Az-geranyl diphosphate including ^1^H and ^31^P NMR spectra (PDF)

## Abbrevation

bactoprenyl phosphate: BP
Az: azido
HPLC: high-performance liquid chromatography
LC–MS: liquid chromatography–mass spectroscopy
Pgl: protein glycosylation
UDP: uridine diphosphate
DiNAcBac: *N*,*N*-diacetylbacillosamine
GlcNAc: *N*-acetylglucosamine
GalNAc: *N*-acetylgalactosamine
HexNAc: *N*-acetyl-hexosamine
SIM: selective ion mode
DBCO: dibenzocyclooctyne
