# Supplementary material for: Chemoenzymatic Preparation of a Campylobacter jejuni Lipid-Linked Heptasaccharide on an Azide-Linked Polyisoprenoid
Source: ACS Omega. 2023 Apr 22;8(17):15790–8. doi: 10.1021/acsomega.3c01657 (PMC10157688; doi:10.1021/acsomega.3c01657)
Supplement: Supplementary file 1 — ao3c01657_si_001.pdf [file ao3c01657_si_001.pdf]

Supporting Information

Chemoenzymatic Preparation of a *Campylobacter jejuni* Lipid-linked Heptasaccharide on an Azide-linked Polyisoprenoid.

Amanda J. Reid,[a] *Katelyn M. Erickson,*[a] *Joseph M. Hazel,*[b,c] *Vinita Lukose,*[d] *Jerry M. Troutman**[a,b]

Nanoscale Science Program,[a] Department of Chemistry,[b] University of North Carolina at Charlotte, 9201 University City Blvd., Charlotte, NC 28223, USA.

Department of Chemistry,[c] The Ohio State University, 281 W Lane Ave, Columbus OH 43210, USA.

Departments of Chemistry and Biology,[d] Massachusetts Institute of Technology, 77 Massachusetts Ave, Cambridge, MA 02139, USA.

Figure S1. Azide isoprenoid products were reacted with DBCO-TAMRA for fluorescence monitoring after respective enzymatic reactions. Benzylazide GPP, BPP, and BP were conjugated to DBCO-TAMRA and product was monitored by HPLC. 3

Figure S2. HPLC analysis of UDP-diNAcBac formation with sequential addition of PglF, E, and D. 4

Figure S3. SDS-PAGE, Ponceau Staining, and Western blot of proteins used in this work. 5

Figure S4. Pgl HexNAc Specificity. 6

Figure S5. 1H NMR and 31P NMR of Az-NPP 7

Figure S6. 1H NMR and 31P NMR of Az-NP 8

Table S1. Primers for wbpP PCR amplification 9

Scheme S1. Benzyleazide analogue synthetic scheme. 10

SI Methods 10

Synthesis of neryl acetate (7) 10

Synthesis of 3,7-Dimethyl-1-acetoxy-2,6-octadien-8-al (2 or 8) 10

Synthesis of 8-N-m-benzyl alcohol-amino-3,7-dimethyl-2,6 octadien-1-ol (3 or 9) 11

Synthesis of 8-N-m-benzyl azido-amino-3,7-dimethyl-2,6 octadien-1-ol (4 or 10) 11

Synthesis of (E, E,)-8-N-m-benzyl azido-amino-3,7-dimethyl-2,6 octadiene diphosphate (5) 11

Synthesis of (Z, E)-8-N-m-benzyl azido-amino-3,7-dimethyl-2,6 octadiene monophosphate (11) 12

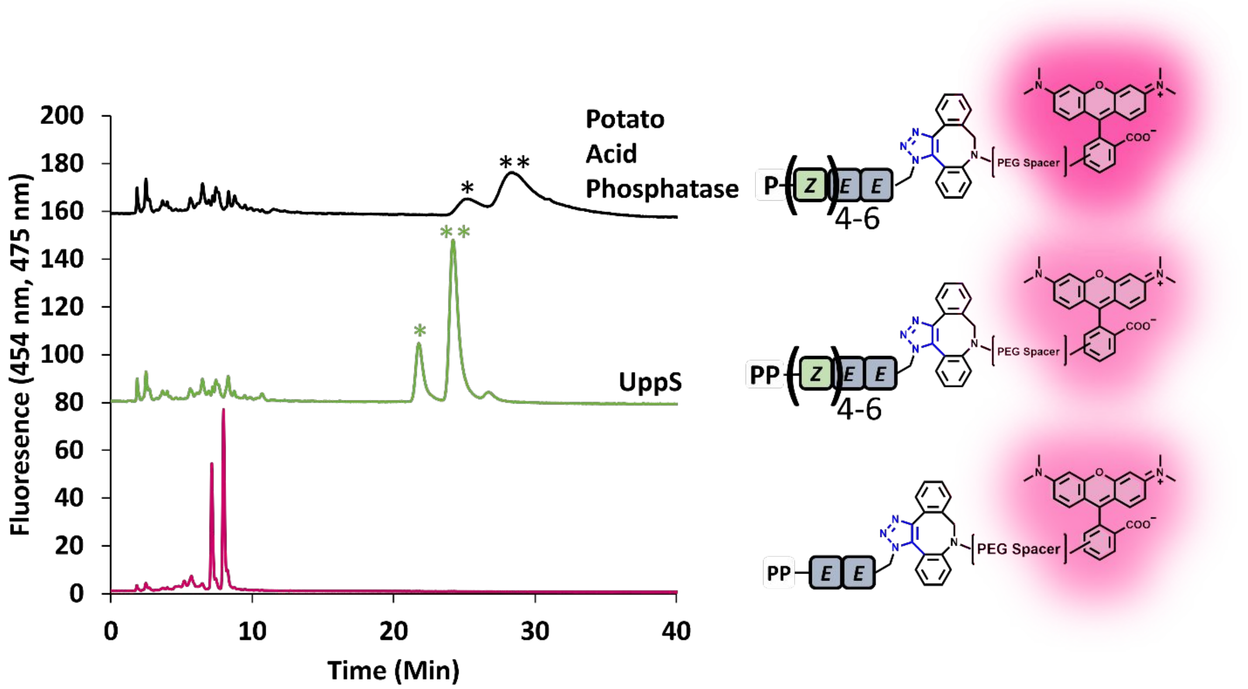

Figure S1. Azide isoprenoid products were reacted with DBCO-TAMRA for fluorescence monitoring after respective enzymatic reactions. Benzylazide GPP, BPP, and BP were conjugated to DBCO-TAMRA and product was monitored by HPLC. Two isomers of TAMRA are apparent in the bottom chromatogram with AzGPP and expected as these are present in commercial sources of DBCO-TAMRA. TAMRA isomers (* and **) in BPP and BP appear to co-elute as each peak represents a different size isoprenoid and peak broadening typical of these products does not allow for resolution observed with the GPP. Samples were blanked with a water injection and the chromatograms were offset along the y-axis by 80 fluorescent units for each sample. Isoprenoid starting material identity was confirmed by LC-MS

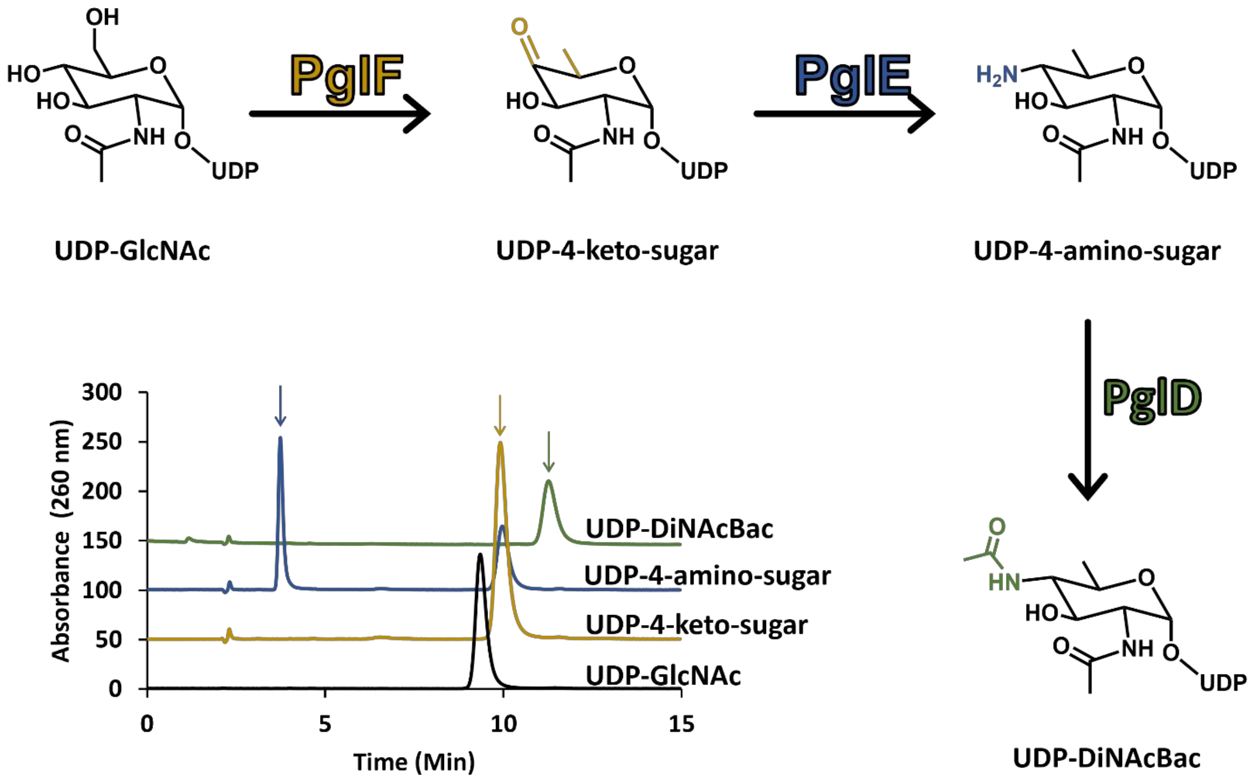

Figure S2. HPLC analysis of UDP-diNAcBac formation with sequential addition of PglF, E, and D. Arrows indicate the elution of respective products. All reactions were performed as single pot reactions from UDP-GlcNAc with successive enzymes. Samples were blanked with a water injection and the chromatograms were offset along the y-axis by 50 absorbance units for each sample.

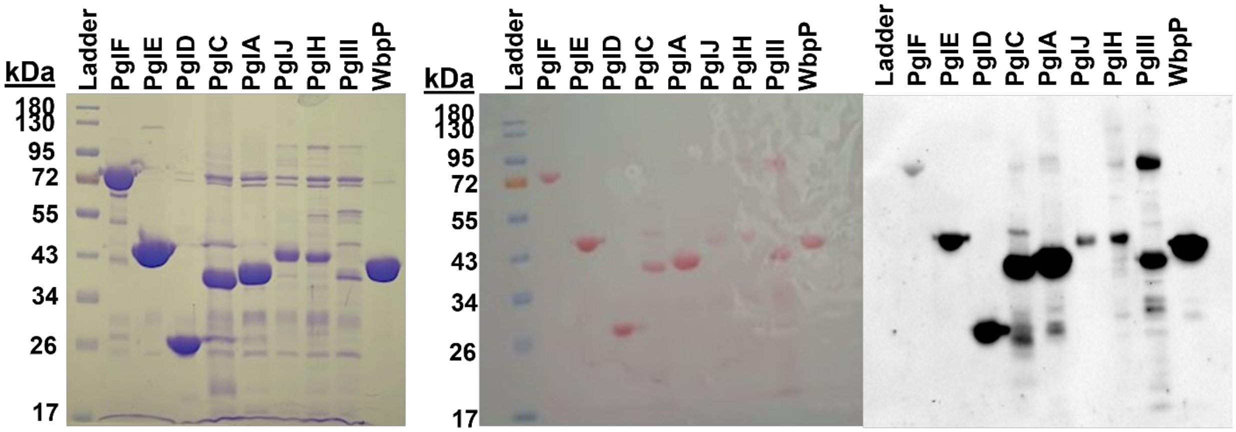

Figure S3. SDS-PAGE, Ponceau Staining, and Western blot of proteins used in this work. SDS-PAGE gels were stained with coomassie. Nitrocellulose blots were stained first in ponceau to confirm successful transfer. Western blots were treated with primary mouse anti-His (1:10,000 dilution) followed by anti-mouse conjugated HRP (1:20,000 dilution). Staining occurred with a chemiluminscent substrate and an exposure time of 30s. All Pgl expressions were carried out in BL21-Star cells and PglI cultures contained 3% ethanol.

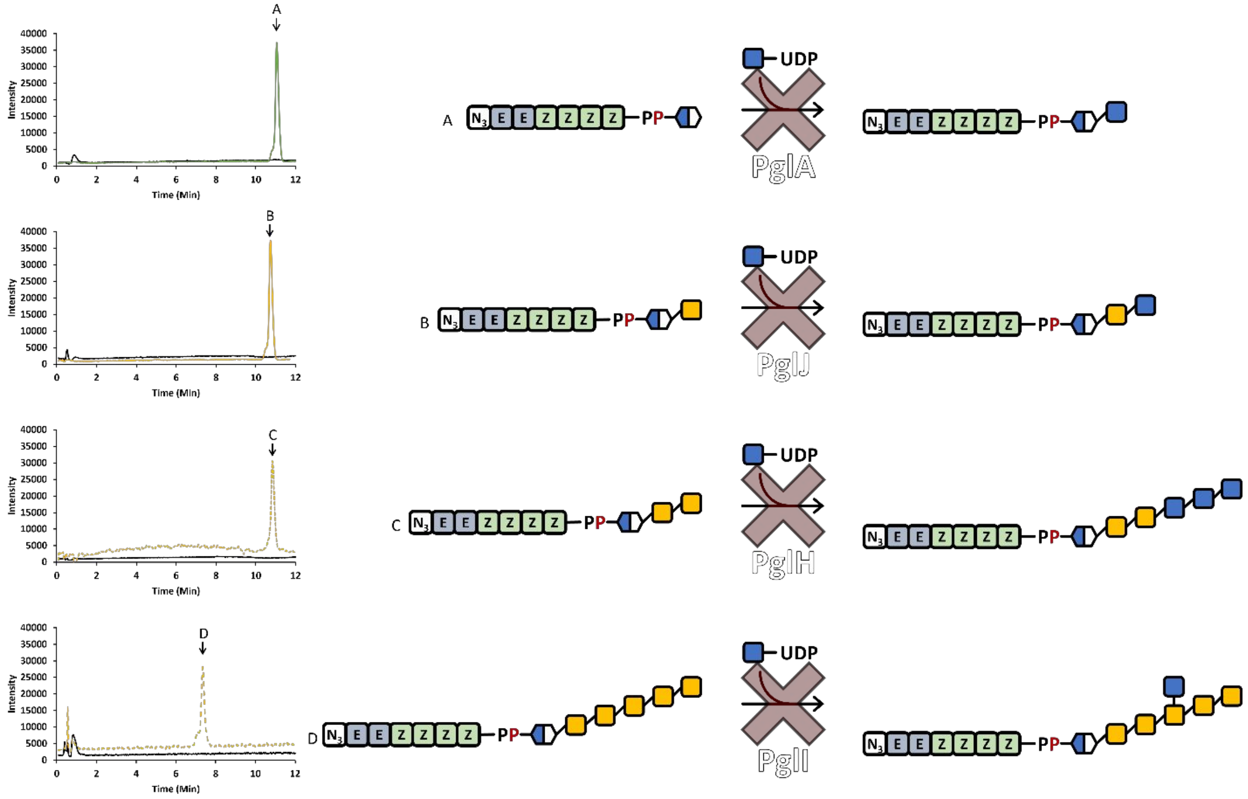

Figure S4. Pgl HexNAc Specificity. Sugar specificity with pgl enzymes and their respective substrate with UDP-GlcNAc in which no transferase activity is observed.

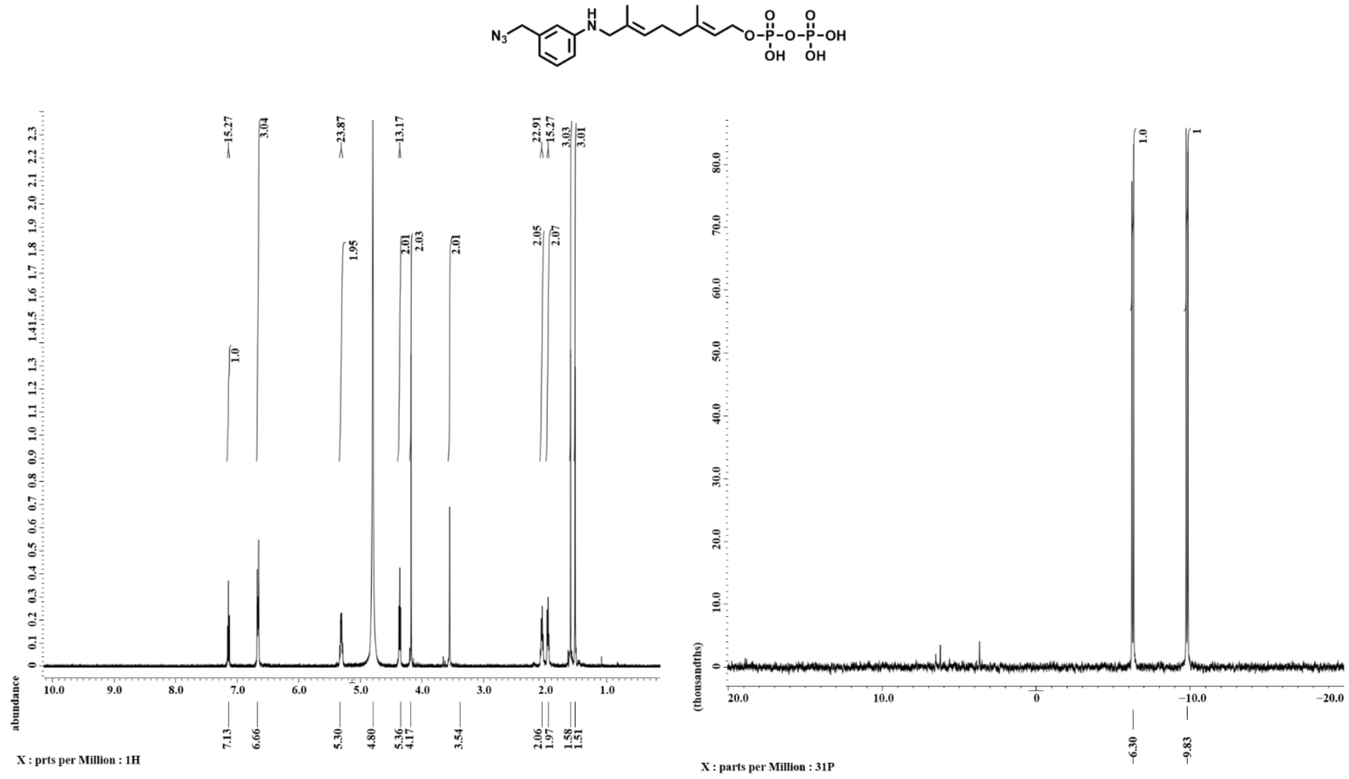

Figure S5. 1H NMR and 31P NMR of Az-NPP

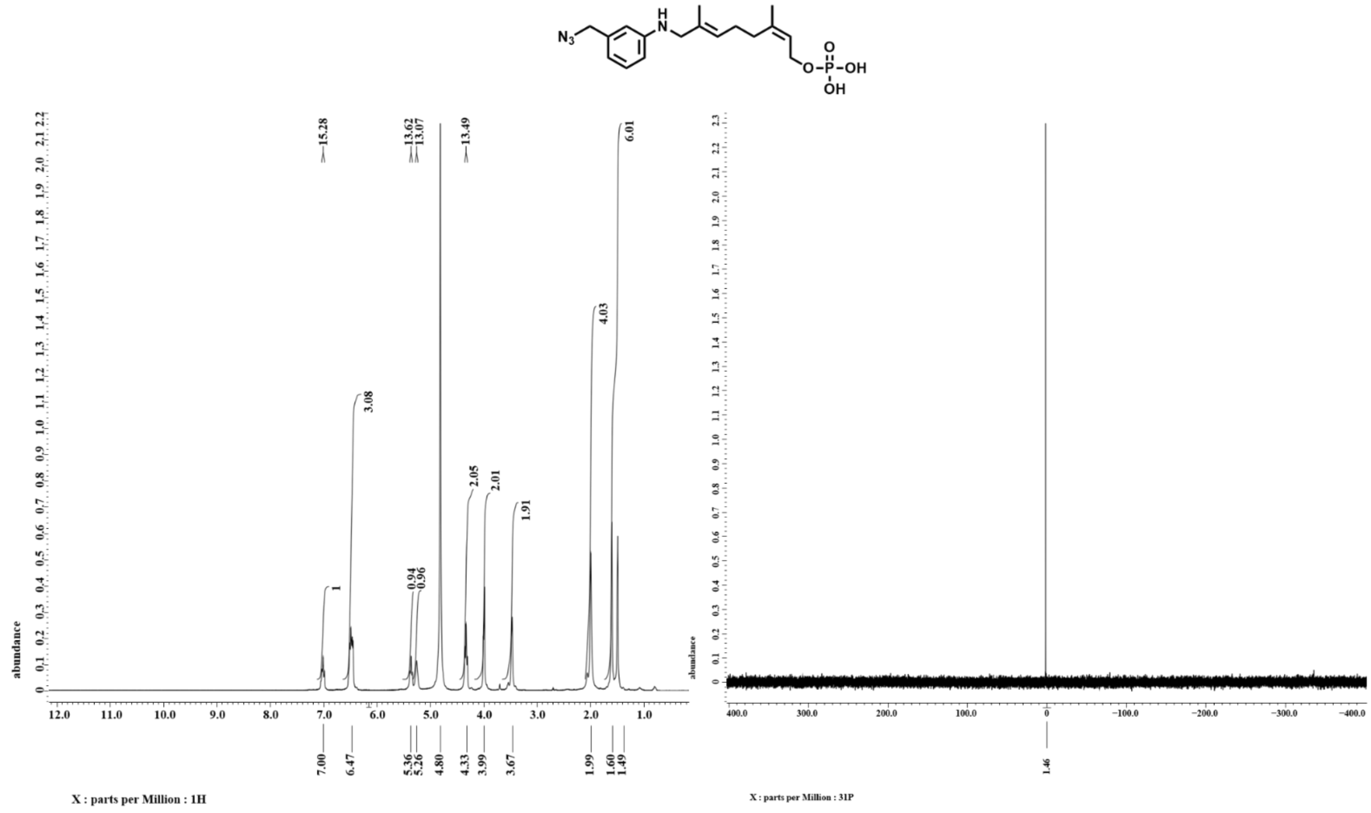

Figure S6. 1H NMR and 31P NMR of Az-NP

Table S1. Primers for wbpP PCR amplification

**Forward**	CGACGGATCCACCAAATACGAAAAAATCCAACAAG
**Reverse**	CCACTCTCGAGTTTTTTATCATTTATAAAGCTTATATACCATGGC

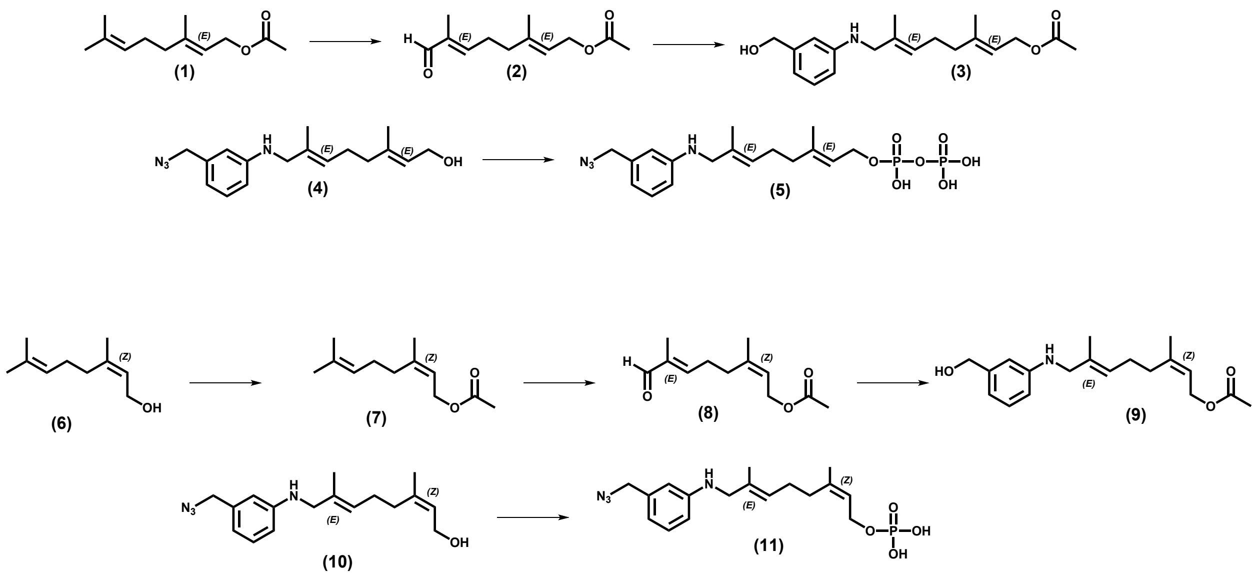

Scheme S1. Benzyleazide analogue synthetic scheme.

SI Methods

The synthesis protocol for benzylazide modified geranyl diphosphate and neryl monophosphate analogues have been adapted from Chehade et al and Labadie et al. 1, 2

Synthesis of neryl acetate (7)

Nerol (5 g, 32.4 mmoles), pyridine (10 mL) and acetic anhydride (10 mL) were added to a round bottom flask. The reaction was allowed to stir at room temperature overnight and then diluted with ether. The crude product was washed with saturated sodium bicarbonate (2x), water, then brine. The organic layer was dried with $MgSO_4$ and the solvent removed by rotary evaporation. The resulting oil was used directly for subsequent allylic oxidation without further purification. ^{1}H NMR (7) (500 MHz, $CDCl_3$, δ): 5.26 (q, J = 7.1, 1H), 5.00 (q, J = 6.2, 1H), 4.46 (d, J = 7.6, 2H), 2.04-1.95 (m, 4H), 1.67 (s, 3H), 1.59 (s, 3H), 1.51 (s, 3H). Yield = quantitative.

Synthesis of 3,7-Dimethyl-1-acetoxy-2,6-octadien-8-al (2 or 8)

SeO_2 0.283 g (2.55 mmols), salicylic acid 0.354 g (2.56 mmols) and 70% tert-butyl hydroperoxide (13.1 mL, 135 mmols) were added to methylene chloride (50 mL) in a round bottom flask and placed in an ice bath. Once the mixture was homogeneous, the acetate (1 or 7) was added (5 g, 25.47 mmols) and the reaction was left stirring overnight. The mixture was diluted with ether and extracted with the following: 5% $NaHCO_3$, saturated $CuSO_4$, saturated $Na_2S_2O_3$ twice, and brine. The organic layer was dried with anhydrous $MgSO_4$ and the solvent was removed. The remaining oil was purified using silica flash chromatography using a 5% (v/v) EtOAc/Hexanes solution. ^{1}H NMR (8) (500 MHz, $CDCl_3$, δ): 9.36 (s, 1H), 6.45 (q, J = 7.3, 1H), 5.40 (q, J = 6.3, 1H), 4.53 (d, J = 7.8, 2H), 2.44 (q, J = 5.6, 2H), 2.29 (q, J = 7.6, 2H), 1.67 (s,

3H), 2.02 (s, 3H), 1.77 (s, 3H), 1.72 (s, 3H). Typical yields of the aldehyde product were between 15-35%.

Synthesis of 8-N-m-benzyl alcohol-amino-3,7-dimethyl-2,6 octadien-1-ol (3 or 9)

A round bottom flask was flame-dried under argon. Once cooled, 50 mL CH_2Cl_2, 1.3 g of 3-aminobenzyl alcohol (10.56 mmols) and 2.0 g (9.5 mmols) of the aldehyde (2 or 8) were added followed by 0.75 mL glacial acetic acid (13.11 mmols) and 3.0 g of $Na(OAc)_3BH$ (14.15 mmols). The mixture was left overnight and extracted with chloroform the following day. The product was purified using 30% EtOAc/Hexanes to isolate the desired product. A contaminate with an R_f value similar to the desired compound was co-purified but did not appear to impact downstream synthesis.

^{1}H NMR (3) (300 MHz, $CDCl_3$, δ): 7.14 (t, *J* = 7.2, 1H), 6.66-6.52 (m, 3H), 5.39 (t, *J* = 6.9, 1H), 5.34 (t, *J* = 6.9, 1H), 4.59 (s, 2H), 4.57 (d, *J* = 7.5, 2H), 3.63 (s, 2H), 2.19-2.08 (m, 4H), 2.03 (s, 3H), 1.72 (s, 3H), 1.68 (s, 3H).

^{1}H NMR (9) (500 MHz, $CDCl_3$, δ): 7.11 (t, *J* = 7.9, 1H), 6.50-6.59 (m, 2H), 6.49 (d, *J* = 5.2, 1H), 5.31 (m, 2H), 4.56 (s, 2H), 4.46 (d, *J* = 3.9, 2H), 4.08 (d, *J* = 5.4, 2H), 3.6 (s, 2H), 2.12 (s, 3H), 2.09-1.98 (m, 4H), 1.75 (s, 3H), 1.67 (s, 3H).

Synthesis of 8-N-m-benzyl azido-amino-3,7-dimethyl-2,6 octadien-1-ol (4 or 10)

The reductive amination products (*3* or *9*) were dissolved in toluene with 2.1 mL of DPPA (2.68 g, 9.74 mmols) and 2.6 g of benzylic alcohol. Geranyl acetate (3 or 9) (8.2 mmols) was added, and the mixture was cooled to 0 °C. Then, 1.5 mL of DBU (1.53 g, 2.91 mmols) was added and allowed to stir on ice for 2 hours. The reaction was left at room temperature overnight and quenched with the addition of an equal volume of water and was diluted with ethyl acetate. The organic layer was dried with $MgSO_4$ and the solvent was removed under reduced pressure. Without further purification, the crude product (theoretical yield of 8.2 mmol) was deacylated in the same flask. The product was resuspended in methanol (40 mL) and 4.7 g of K_2CO_3 (34 mmol) dissolved in 5 mL of water and was left stirring at room temperature overnight. Pure 8-N-m-benzyl alcohol-amino-3,7-dimethyl-2,6 octadien-1-ol was obtained with 40% EtOAc/Hexanes (R_f = 0.3 in 30% EtOAc/Hexanes).

^{1}H NMR (4) (300 MHz, $CDCl_3$, δ): 7.14 (t, *J* = 7.8, 1H), 6.60-6.51 (m, 3H), 5.36 (q, *J* = 6.5, 2H), 4.18 (s, 2H), 4.08 (d, *J* = 7.0, 2H), 3.60 (s, 2H), 2.14-2.02 (m, 4H), 1.64 (m, 6H).

^{1}H NMR (4) (300 MHz, $CDCl_3$, δ): 7.14 (t, *J* = 7.8, 1H), 6.60-6.51 (m, 3H), 5.36 (q, *J* = 6.5, 2H), 4.18 (s, 2H), 4.08 (d, *J* = 7.0, 2H), 3.60 (s, 2H), 2.14-2.02 (m, 4H), 1.64 (m, 6H).

Synthesis of (E, E,)-8-N-m-benzyl azido-amino-3,7-dimethyl-2,6 octadiene diphosphate (5)

The benzylazide geranyl alcohol (*4*) was brominated followed by subsequent diphosphorylation. 8-N-m- benzyl alcohol-amino-3,7-dimethyl-2,6 octadien-1-ol (333 µmol) was added to a flame dried flask under argon as 1 mL of a 100 mg/mL solution in methylene chloride. From a 1 M solution, 0.17 mL of PBr_3 in Cl_3CH (170 µmol) was then added to the flask, without further addition of solvent. Bromination occurred instantaneously. Without further

purification, 1.8 mL of tris tetra n-butyl ammonium diphosphate in acetonitrile (0.5 mg/mL) was added and the reaction (999 μmol) was left at room temperature for 2h. The solvent was removed under reduced pressure without heat. The resulting viscous solution was resuspended in minimal 25 mM ammonium bicarbonate (generally less than 0.5 mL). The crude reaction was then placed directly on NH_4^+ charged cation exchange resin and eluted with the same buffer. The compound was then frozen and lyophilized prior to purification by HPLC. ^{1}H NMR of (5) (500 MHz, D_2O, δ): 7.13 (t, J = 7.64, 1H), 6.66 (m, 3H), 5.30 (q, J = 5.97, 2H), 4.36 (t, J = 6.59, 2H), 4.17 (s, 2H), 3.54 (s, 2H), 2.06 (m, 2H) 1.97 (m, 2H), 1.58 (s, 3H), 1.51 (s, 3H). ^{31}P (D_2O, δ): -6.30 (1P), -9.83 (1P). Expected m/z 459.1, obtained 459.3 m/z; Extinction coefficient ε= 4,345 M^{-1} cm^{-1} at 260 nm; Typical yield =25-35%

Synthesis of (Z, E)-8-N-m-benzyl azido-amino-3,7-dimethyl-2,6 octadiene monophosphate (11)

To a flame dried flask, 1 mL of a 100 mg/mL solution in CH_2Cl_2 of (*10*) (333 μmol) and excess trichloroacetonitrile (217 μL, 2.2 mmol) was added under argon. Solid tetra-n-butylammonium dihyrogen phosphate (226 mg, 6.7 mmol) was then added, which was prepared from the lyophilized product after titrating phosphoric acid.[3] The mixture was stirred for 10 min and the solvent removed. The viscous solution was resuspended in an equilibrated mixture of THF (2 mL) with 25% (v/v) ammonium hydroxide (0.2 mL) and stirred for 30 min. Next, 5 mL of a toluene:methanol (1:1) was added for an additional 20 min. The resulting precipitate was removed by filtration and the solvent removed. The crude reaction was resuspended in minimal 25 mM ammonium bicarbonate with 10% isopropanol (generally less than 0.5 mL) and placed directly on NH_4^+ charged cation exchange resin and eluted. The compound was then frozen and lyophilized prior to purification by HPLC. Purified fractions were dried under vacuum. ^{1}H NMR of (11) (500 MHz, D_2O, δ): 7.00 (t, J = 7.64 1H), 6.47 (m, 3H), 5.36 (t, J = 6.81, 2H), 5.26 (t, J = 6.54, 1H), 4.33 (t, J = 6.75, 2H), 3.99 (s, 2H), 3.67 (s, 2H), 1.99 (s, 4H), 1.60 (s, 3H), 1.49 (s, 3H). ^{31}P (D_2O, δ): 1.46 (1P). Expected m/z 379.2, obtained 379.0 m/z; Typical yield ~ 50%

1. Chehade, K. A. H.; Andres, D. A.; Morimoto, H.; Spielmann, H. P., Design and synthesis of a transferable farnesyl pyrophosphate analogue to Ras by protein farnesyltransferase. *J Org Chem* **2000,** *65* (10), 3027-3033.
2. Labadie, G. R.; Viswanathan, R.; Poulter, C. D., Farnesyl diphosphate analogues with omega-bioorthogonal azide and alkyne functional groups for protein farnesyl transferase-catalyzed Ligation reactions. *J Org Chem* **2007,** *72* (24), 9291-9297.
3. Xu, H. J.; Zhao, Y. Q.; Zhou, X. F., Palladium-catalyzed Heck reaction of aryl chlorides under mild conditions promoted by organic ionic bases. *J Org Chem* **2011,** *76* (19), 8036-41.
